# Effects of Heat and Momentum Gain Differentiation during Gas Detonation Spraying of FeAl Powder Particles into the Water

**DOI:** 10.3390/ma14237443

**Published:** 2021-12-04

**Authors:** Cezary Senderowski, Andrzej J. Panas, Bartosz Fikus, Dariusz Zasada, Mateusz Kopec, Kostyantyn V. Korytchenko

**Affiliations:** 1 Department of Materials Technology and Machinery, University of Warmia and Mazury, 10-719 Olsztyn, Poland; 2 Faculty of Mechatronics, Armament and Aerospace, Military University of Technology, 00-908 Warsaw, Poland; andrzej.panas@wat.edu.pl (A.J.P.); bartosz.fikus@wat.edu.pl (B.F.); 3 Faculty of Advanced Technologies and Chemistry, Military University of Technology, 00-908 Warsaw, Poland; dariusz.zasada@wat.edu.pl; 4 Institute of Fundamental Technological Research, Polish Academy of Sciences, 02-106 Warsaw, Poland; mkopec@ippt.pan.pl; 5 Department of Mechanical Engineering, Imperial College London, London SW7 2AZ, UK; 6 Kharkiv Polytechnic Institute, National Technical University, 61-002 Kharkiv, Ukraine; korytchenko_kv@ukr.net

**Keywords:** D-gun spraying, FeAl intermetallic powder, gas detonation flux, heat transfer, two-phase metallization stream, particle thermal history

## Abstract

In this paper, dynamic interactions between the FeAl particles and the gaseous detonation stream during supersonic D-gun spraying (DGS) conditions into the water are discussed in detail. Analytical and numerical models for the prediction of momentum and complex heat exchange, that includes radiative effects of heat transfer between the FeAl particle and the D-gun barrel wall and phase transformations due to melting and evaporation of the FeAl phase, are analyzed. Phase transformations identified during the DGS process impose the limit of FeAl grain size, which is required to maintain a solid state of aggregation during a collision with the substrate material. The identification of the characteristic time values for particle acceleration in the supersonic gas detonation flux, their convective heating and heat diffusion enable to assess the aggregation state of FeAl particles sprayed into water under certain DGS conditions.

## 1. Introduction

A unique feature of DGS technology is very high kinetic energy and thermal energy accumulated in the two-phase (gas and powder) metallization stream (that cause a large volumetric strain of the powder particles heated to very high temperatures), which changes non-monotonically with the dynamics of the detonation gas flow stream [1,2,3,4,5,6].

Parameters that affect a detonation wave include the gas velocity (D), its pressure (p), density (*ρ*) and temperature (T). These parameters can be considered within the frame of hydrodynamic theory and unidimensional stationary detonation (Zeldowicz–von Neumann–Doering (ZND) theory) [7,8,9,10]. According to this theory, the detonation wave consists of the shock wave (FU), the chemical reaction zone and the detonation products zone.

**Figure 1:**
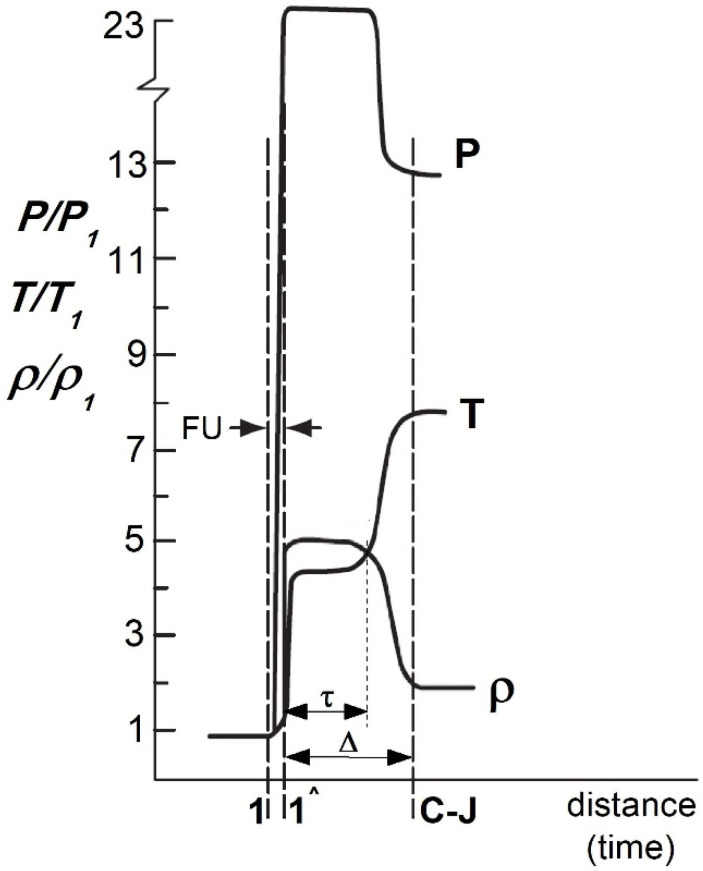
Change of gas thermodynamic parameters in the detonation wave area where: FU—shock wave, C-J—Chapman–Jouget plane.

The shock wave is typically reduced to the (1-1) surface of high discontinuity in the pressure (p), density (*ρ*) and temperature (T) of the gas blend at the moment of detonation (Figure 1) [8].

The hypothetically infinitesimal chemical reaction zone is determined by the duration of the chemical reaction, that follows the wave and the velocity of detonation wave. Such velocity is constant and could be reached only after covering some distance (∆) from the ignition point—in the Chapman–Jouget (C-J) plane.

Consequently, the system changes into the defined detonation wave, characterized by the detonation products, where temperature, density, pressure, velocity and chemical composition are dependent on the type of the gas blend.

Out of a series of interdependent conditions accompanying gas detonation, the composition of the explosive mixture has the greatest impact on the structure of the detonation wave, and thus on the value of thermodynamic parameters and the flow velocity of the two-phase metallization stream. It is mainly characterized by the type of working gas fuel and its share in relation to the oxidant (oxygen or air) and the method of diluting the explosive mixture by additionally introducing an inert gas as a diluter gas that delay the detonation. The analysis of the gas detonation mechanism and simulation data that is based on Euler’s equations and ZND model, a strong relationship between the stoichiometry coefficient (*λ*) and the type of oxidant used with the detonation wave structure could be observed [2,4,9,10,11,12]. As already mentioned, this structure is determined by the thickness of the chemical reaction zone and the time of their course, which depends on the type of operating fuel.

The analysis of the relationship between the type and composition of the explosive mixtures and the detonation wave structure shows that the thickness of the chemical reaction zone and the time of its course reach the minimum values for approximately stoichiometric compositions regardless of the type of fuel and the accompanying oxidant [8]. The dimensions of the chemical reaction zone immediately downstream of the FU are always smaller for oxygen-fuel mixtures as compared to mixtures with air as the oxidant. The presence of nitrogen in the composition of the explosive detonation mixture with air (and also often intentionally introduced as a phlegmatizer to the fuel-oxygen mixture) limits the level of temperature and pressure that activate the processes of chemical reactions in detonation combustion after FU passing.

However, regardless of the type of demolition mixture used, the crossing of the detonation wave by the (1-1) surface results in the pressure increase (up to several atmospheres) followed by temperature increase (to a couple of thousand Kelvin above the critical temperature in which the gas enters the chemical reaction—Figure 1) [8,10,11,12,13,14]. Such phenomena are associated with a huge release of heat energy, with a simultaneous drop in pressure and density of the gas stream, which reaches the speed of sound at a Chapman–Jouget plane. Simultaneously, a multi expansion of the gaseous products, whose flow is being slowed due to the high pressure prevailing at the front of the moving concentrated shock wave, occurred (C-J plane—Figure 1). Such expansion results in rapid movement of the detonation wave toward the muzzle. As it reaches the open end of the barrel, the expansion of the gaseous detonation products at a supersonic speed with a series of successive stages of compression and expansion of gas products occurred [11,12].

The wave structure of the metallization stream’s supersonic flow with the formation of Mach disks is relatively quickly extinguished by mutual friction of hot gas molecules with air molecules from the atmosphere induced by a turbulent shear layer of viscous damping [8]. However, damped oscillations of velocity and temperature of gaseous products, directly after their exit from the barrel of DGS (until the stream reaches the subsonic speed), also exert a significant influence on the final velocity and the degree of heating of the powder particles at the time of impact with the substrate material. This applies particularly to small particles with low inertia, which achieve the highest speed but are quickly slowed down [8,11,12].

In the final phase of the metallization stream flow, the thermal energy of the powder particles directly involved in the forming of the coating structure is determined by the dynamic pressure pulse at the moment of impact with the substrate, which induces significant changes in the temperature of the adhesion-diffusion junction. The literature shows, that for nickel powder feedstock with particle velocity greater than 500 m/s, a temperature increase results from the collision with the substrate. Such an increase of up to 300 °C [15,16] may in turn lead to the melting of the strongly softened powder particles. 

Both the significant volume of the liquid phase (more than 10% vol.) resulting from the melting of the powder particles upon collision with the substrate during coating deposition, and the under-heating of the particles (powder temperature below 0.9 Tm—absolute melting point) result in a relatively high coating porosity of 4–6% [15]. The FeAl phase equilibrium system shows that the high-temperature FeAl solid solution (the composition of which is close to stochiometric) is stable at temperatures up to 1310 °C [8,17,18]. This fact is extremely important from a technological point of view. It can be hypothetically assumed that the lack of melting of the feedstock material during the DGS process enables the phase and chemical composition of the FeAl feedstock powder material to be maintained [19,20,21,22]. The fundamental problem raised during the optimization of the DGS process parameters is to ensure that the particles of the feedstock powder would retain the solid state while reaching their softening temperature. That enables the softened FeAl particles to undergo plastic deformation upon impact onto the substrate and geometrically transform them into the FeAl coating with unique properties [23,24,25]. Thus, the main aim of this work was to obtain data describing the process of heating and cooling of FeAl powder particles working under the dynamically changing conditions of the DGS spraying cycle. On the other hand, the work has been performed to develop and experimentally verify an analytical model of heat absorption by FeAl intermetallic powder particles in a flux of gaseous products during detonation combustion that occur during the D-gun spraying process of FeAl powder particles into the water. The analytical model of momentum and convective heat transfer between the FeAl particle and the gas flowing around them allowed for both, a precise description of such important phenomena during the gas detonation process, and a determination of the optimized conditions of FeAl particles thermal state. The model enables an estimation of the value of energy acquired by FeAl powder particles with different sizes in order to maintain the solid state in the D-gun spraying process. Thus, it is possible to determine the known size distribution of feedstock powder particles, the optimal parameters of DGS spraying at which FeAl powder particles reach the substrate in the solid state but highly softened.

On the contrary to known thermal spraying techniques [26], the detonation spraying procedure is characterized by a much higher complexity level [27,28]. It is because unstable gas flow conditions with high temperature and pressure gradients and high rates of local temporal DGS parameters change during the process (comp. e.g., [7,9,10,11,12,13,14]). During the analysis of the heat, momentum and mass transfer phenomena, the problems of coupling of various processes and their non-equilibrium character occurred. Therefore, such phenomena were subsequently confirmed by the assessment of heating degree of the FeAl powder particles under specific DGS spraying conditions into the water. It should be mentioned that the main work concentrated on demonstrating that for a given powder particle size distribution, only part of the particles would be melted. For this purpose, the parameters of the gas flow detonation were determined and the effects of heat and momentum exchange between the carrier flue gases and the powder particles were estimated.

## 2. Technological Conditions in DGS Process

Gas detonation spraying was performed at the E.O. Paton Electric Welding Institute of Ukrainian Academy of Sciences, Kiev by using the ‘‘Perun-S’’ detonation system (National Academy of Sciences of Ukraine, Kiev, Ukraine) (barrel length of 590 mm and 23 mm diameter).

An elementary working cycle of the DGS process starts with filling the channel with a specific explosive mixture. Subsequently, a certain portion (0.2–0.3 g) of the feedstock powder material is cyclically, axisymmetrically fed to the power injection position (PIP) by a carrier gas (i.e., nitrogen or air). PIP is the location of the powder in the barrel gun at the time of detonation. After such an injection, an electric spark with a constant frequency of 6.66 Hz is used to initiate detonation. Subsequently, the barrel line is rinsed with inert gas (nitrogen) after each unit cycle to prevent an excessive temperature increase of the barrel and substrate material, which during spraying does not exceed 100–150 °C.

In the presented research, the analysis of FeAl powder particles heating was compared to the unit operating cycle of the “Perun-S” gun during the actual DGS process of Fe40Al at.% powder particles into water, with the parameters presented in Table 1.

**Figure 2:**
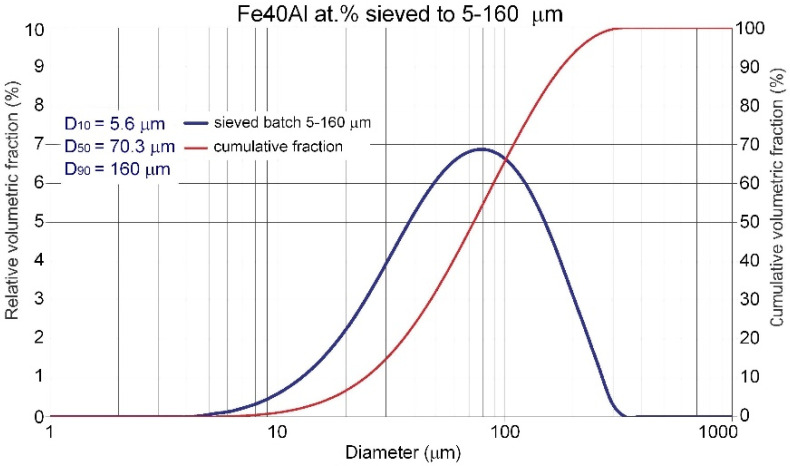
Particle size distribution of the Fe40Al at.% feedstock powder material as declared by the supplier.

Commercial Fe40Al at.% powder with a wide particle size range from 5 µm to 160 µm (Figure 2), produced by using the VIGA method (vacuum inert gas atomization) in the CEA “LERMPS laboratory” in Grenoble, France, was used in this study. The IPS UA analyzer with an infrared diode (Kamika Instruments Company, Warsaw, Poland) was used to assess the particle size distribution of the FeAl powder particles. This analyzer enabled identification of the particle size with the additional possibility of precise counting in specific, declared size ranges. Scanning electron microscopy with X-ray microanalysis (Philips XL30/LaB6-DX4i-EDAX, Eindhoven, The Netherlands) was used to assess the degree of structural homogeneity (morphology and particle size of the FeAl powder in its as-received state and after DGS spraying into water).

### Model Flow Parameters in DGS Process

The elementary work cycle of “Perun-S” lasts only 150 ms and it is repeated cyclically with a frequency of 6.66 Hz. In such a cycle a few important issues are considered:

- Thermodynamic properties and flow kinetics of gaseous detonation products, dependent mainly on the composition and the flow rate of the explosive mixture;
- Physicochemical and structural properties of the powder material, related to the average size, morphology, phase composition and the melting, evaporation and sublimation point of the powder particles.

Taking into account the frequency of shots of 6.66 Hz for the “Perun-S” gun during spraying FeAl coatings, the molar concentration of individual components of the gaseous explosive mixture at one working cycle of operation corresponds to the values presented in Table 2.

Calculation of the thermal parameters of the gas phase was performed using the TIGER 9.0 thermochemical code developed at Lawrence Livermore National Laboratory (LLNL) that considers the assumption of local chemical equilibrium for the detonation combustion conditions of a given propane explosive mixture with oxygen and air. Such code enables thermodynamic calculations for non-ideal, heterogeneous chemical systems of known atomic composition, containing gases and condensed phases (liquids and solid particles), described by the adopted gas state equations in detonation conditions [8]. Detonation parameters and the thermodynamic state of combustion products at the time of the detonation explosion of the mixture (in the Chapman–Jouget plane) were presented in Table 3. Weight and molar fractions of combustion products were calculated from the balance of chemical equilibrium using the libraries with free enthalpy values for specific reaction products implemented in the TIGER program (Table 4).

In order to estimate the heat transfer parameters, it is necessary to determine both the thermo-kinetic properties of the explosive gas mixture under the applied detonation combustion conditions (presented above) and the thermo-physical properties of the FeAl type intermetallic powder material.

**Figure 3:**
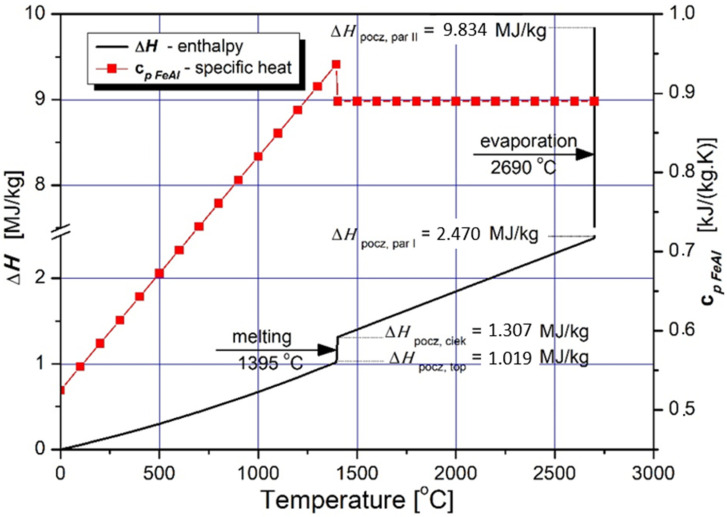
Dependence of the specific enthalpy of FeAl on the temperature (continuous line) obtained from model calculated dependence of the specific heat at the constant pressure with consideration of the value of enthalpy of phase changes of melting and evaporation (in accordance with Table 5).

The characteristics of specific heat changes of the Fe40Al at.% feedstock powder material with the accompanying course of specific enthalpy changes as a function of temperature, were considered as presented in Table 5 and Figure 3 [8,14,29].

**Figure 4:**
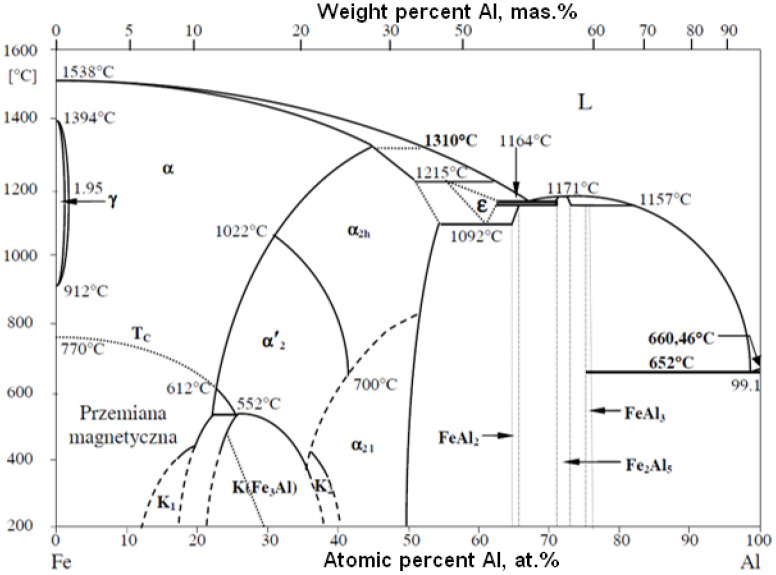
FeAl equilibrium system.

Specific enthalpy is the main parameter that determines the amount of energy required for isobaric heating, melting and evaporating the powder feedstock material as a function of temperature. Despite the fact that the FeAl phase is stable up to 1310 °C (Figure 4), the first temperature range was selected to be from 0 °C to 1395 °C, which is the model liquidus temperature for the Fe40Al at.% alloy. The second range from 1395 °C to the conventional evaporation temperature of 2690 °C was determined on the basis of the arithmetic mean of the evaporation temperatures of Al and Fe [30,31,32,33,34,35].

## 3. Assessment of Heat and Momentum Transfer Effects on FeAl Particle Thermodynamic State

The analysis of complex heat, momentum and mass transfer phenomena shaping the DGS spraying of FeAl particles was performed at two stages. First, through analytical considerations, it was proved that there is a certain particle diameter limit above which particles maintain their solid state [8,29]. Second, dynamics and thermal history of FeAl powder particles were analyzed through numerical calculations [14].

Details concerning modelling heat and momentum transfer between particle and detonation gases in a multiphase flow were provided in [8,14,29].

In general, the theoretical model for the momentum and convective heat exchange between the FeAl particle and the gaseous phase was considered to study effects such as particle melting, evaporation/sublimation, heat conduction in the volume of the FeAl particle and the radiation contribution to heat exchange between the FeAl particle and the walls of the detonation gun barrel. The analysis omitted the multi-phase structure of FeAl particles resulting from the presence of oxide phases formed in situ under DGS spraying conditions and the porosity of the powder particles. The sprayed FeAl particles were assumed as sphere shaped with an equivalent diameter, d. Moreover, the volume occupied by the powder particles was negligible in relation to the volume of the detonation gun barrel, so the presence of particles introduced in the amount of up to 0.25 g during the unit operation cycle of the gun, did not significantly affect the propagation of the detonation wave.

The dynamics of the flow and heat exchange process between the FeAl powder particle and the detonation wave, and the subsequent stream of gaseous products of the detonation combustion, make the particles heat up in a gas of variable velocity and with different temperatures. However, once the shock wave front (FD) has passed through, the parameters of the thermodynamic state of the gaseous detonation products in the vicinity of a single particle can be considered as homogeneous.

In the theoretical modelling, the ability of the powder particle to maintain a solid state at the moment of collision with the base material was based on the comparison of the modeled time values (τ_V_, τ_T_, and τ_a_)—with respect to the approximate time of the FeAl particle exceeding the limit of the detonation combustion zone initiated by the shock wave (τ_A_) and time of flight (τ_B_)—from the point of insertion in the gun barrel (PIP) to the base material (water).

Where (τ_V_, τ_T_, and τ_a_) mean respectively:

(τ_V_)—particle acceleration time;
(τ_T_)—particle convection heating time;
(τ_a_)—temperature equalization time in the particle volume (heat diffusion).

**Figure 5:**
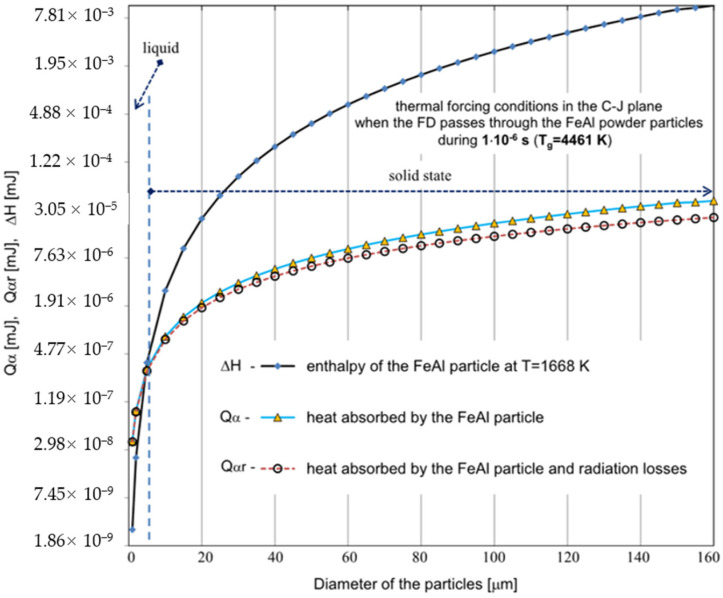
Comparison of the calculated values for the thermal energy Q_α_ absorbed by the powder particles with the enthalpy of the FeAl alloy in the solid state at the melting temperature ∆H, for different values of the particle diameter ranging from 1 µm to 160 µm. For the calculations, the parameters corresponding to the temperature of the gaseous medium of T = 4461 K during its interaction time of τ_A_ = 1 × 10^−6^ s were adopted (the dashed line represents the results of the Q_αr_ calculations considering radiation losses).

The state of the FeAl particle was determined by comparing the enthalpy of the phase transition of melting and evaporation of the FeAl phase with the amount of energy (Figure 5) that the particle was able to absorb from the surrounding gas in the time from the transition of the detonation wave to the impact on the substrate. The main assumption involves the particle dwelling time in the interaction zone of gaseous products in the detonation combustion. The average velocity of FeAl powder particles, measured experimentally at the moment of their collision with the base material, for the technological conditions of DGS spraying in question, was approx. 730 m/s [8]. Thus, it was established that the total flight time of the powder particles to reach the “water level” in the tank, located 110 mm from the muzzle of the “Perun-S” gun, was 5.82 × 10^−4^ s, including the time of the particles remaining in the barrel of the “Perun-S” gun – 4.32 × 10^−4^ s that underestimates the real time of particle exposition to combustion gases.

Generally, the duration of the gas detonation process is very short and depends on the composition of the explosive mixture. According to the literature [2,9,10,11,12,13,14], the time of the detonation wave interaction in the C-J plane (τ_C-J_) and the time of powder particle flight in the stream of gaseous products of detonation combustion (τ) are respectively: τ_C-J_ = 10^−7^ − 10^−5^ s and τ = 10^−4^ − 10^−3^ s.

**Figure 6:**
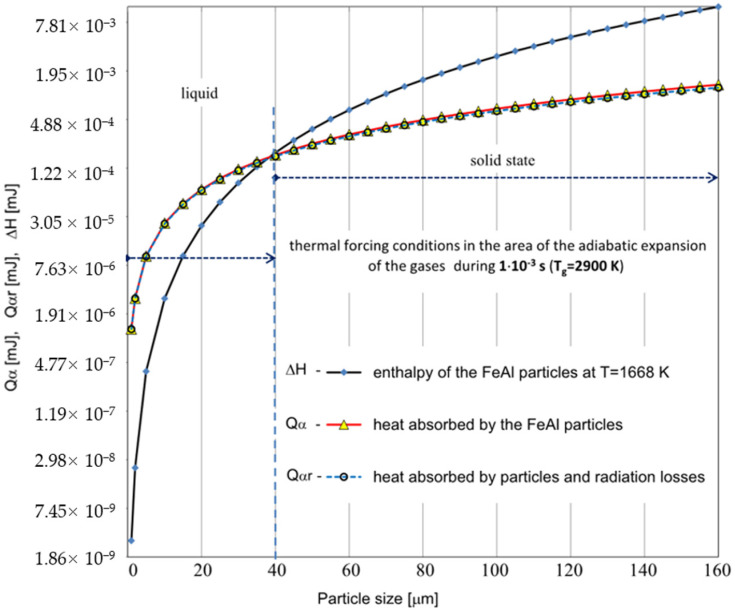
Comparison of the calculated values for the thermal energy Q_α_ absorbed by the powder particles with the enthalpy of the FeAl alloy in the solid state at the melting temperature ∆H, for different values of the particle diameter ranging from 1 µm to 160 µm. For the calculations, the parameters corresponding to the temperature of the gaseous medium of T = 2900 K during its interaction time of τ_A_ = 1 × 10^−3^ s were adopted (the dashed line represents the results of the Q_αr_ calculations considering radiation losses).

Establishing specific velocities of the DGS process, the effectiveness of a specific method of heat exchange between the particle and the surrounding gas was assessed in terms of the heating of FeAl powder particles under the conditions of the DGS experiment (Figure 5 and Figure 6).

Based on the analytical simplified modeling results, it can be concluded that particles with a diameter already above 40 µm will not be able to absorb enough heat to melt the FeAl material. This is evidenced by the analysis of the graphs shown in Figure 5 and Figure 6—illustrating the comparison of the thermal energy Q absorbed by the particles of the powder charge with the enthalpy of the FeAl material in the solid state, at the melting temperature (ΔH), for the particle diameter ranging from 1 µm to 160 µm.

Whereby, under the thermal forcing conditions generated in the CJ plane, when the FD passes through the powder particles, the amount of thermal energy absorbed by the particle is limited by the short residence time of the particle in the detonation wave zone, and thus particles up to 5 µm in diameter are melted (Figure 5). In this case, the greater influence of radiation effects can be observed during the heat transfer between the FeAl particle and the walls of the detonation gun.

Under the thermal forcing conditions generated in the C-J plane, when FD passes through the powder particles, the particle velocity changes are much faster than the particle temperature changes caused by its convective heating. Thus, despite the negligibly low temperature equalization resistances in the FeAl particle (i.e., low heat conduction resistances), it will not obtain the gas temperature in its entire volume during the passage of the detonation wave, because the enthalpy of the particle increases by convective heat supply through its surface. Under these conditions, the amount of thermal energy absorbed by the particle is limited by the short residence time of the particle in the detonation wave zone.

As the powder particles accelerate the fastest when passing through the FD and then strive to equalize their velocity with the stream of gaseous products of detonation combustion, their main heating takes place in a slower flow of the gas stream (approx. 10–3 s) with lower values of the heat transfer coefficient. During the flow in the expansion stage of the gaseous products, the particle velocity gradually equalizes with the gas velocity. Due to the polydispersity of FeAl powder particles in the particle size range of 1–160 µm, they are characterized by a different flow velocity, spreading in the stream of hot gases, along the length of the barrel gun. As the biphasic metallization stream spreads, the amount of heat transferred to the finer FeAl particles remaining in the “tail of the stream” increases due to their lower flight speed and longer residence time in the gas stream. These particles, due to their greater inertia, experience less acceleration in the first phase of flight, when the detonation wave passes through them. Moreover, as the particle velocity equalizes with the gas velocity, the heat transfer coefficient decreases. At the same time, the corresponding convective heating time lengthens.

Hence, with the adopted assumptions model, the exact determination of the limit diameter of non-remelting particles is a complex issue. However, it is undisputed that such a boarder limit exists and further depends on the time that the particles spent in gaseous stream of the detonation products.

In order to verify the results and to further obtain a more precise estimation, the numerical calculations were performed using the Ansys Fluent CFD 2019 software, as described in [14].

Similar to the presented analytical modelling, the whole analysis was restricted to one single cycle of gas detonation and particle propulsion. Because the crucial stage of propulsion takes place in the interval of gas outflow from the barrel, therefore the numerical simulation was terminated when the largest particle reached the base surface, i.e., water level. For the momentum transfer and heat exchange between the gas phase and the powder particles, the same formulas as in the previous analysis were applied. However, the combustion reactions were modelled independently in order to match the parameters obtained from the TIGER code in C-J plane (as shown in Table 3). As a result of the conducted simulations, the spatial distributions of the gas physical parameters, as well as the time history of the particles’ state and motion, were obtained. Comparison of the effects of theoretical modeling with numerical experiments suggests an acceptable discrepancy of the results. The comparison of theoretical modeling with numerical experiments indicates an acceptable discrepancy in velocity results of 920 and 710 m/s, for 10 and 20 µm diameter particles, respectively [14], which is consistent with the velocity measurement in the DGS experiment. In order to demonstrate the effect of heating during the whole DGS process, both the temperature changes in the surface of FeAl particles and the radial temperature distribution were obtained from the numerical analysis. The results were subsequently related to the heat transfer coefficient evolution as a function of time, which is extremely high for small particles exposed to high temperature, while undergoing the highest acceleration in the first phase, during the detonation wave interaction.

It was concluded that the thermal diffusivity of FeAl powder particles up to 60 µm in diameter (mainly determined by the thermophysical properties of the FeAl phase) did not constitute any barrier to reaching the temperature in the particle volume in line with temperature changes on its surface. Thus, basically the FeAl powder particles heat up in the gas stream evenly throughout their entire volume, regardless of their size.

**Figure 7:**
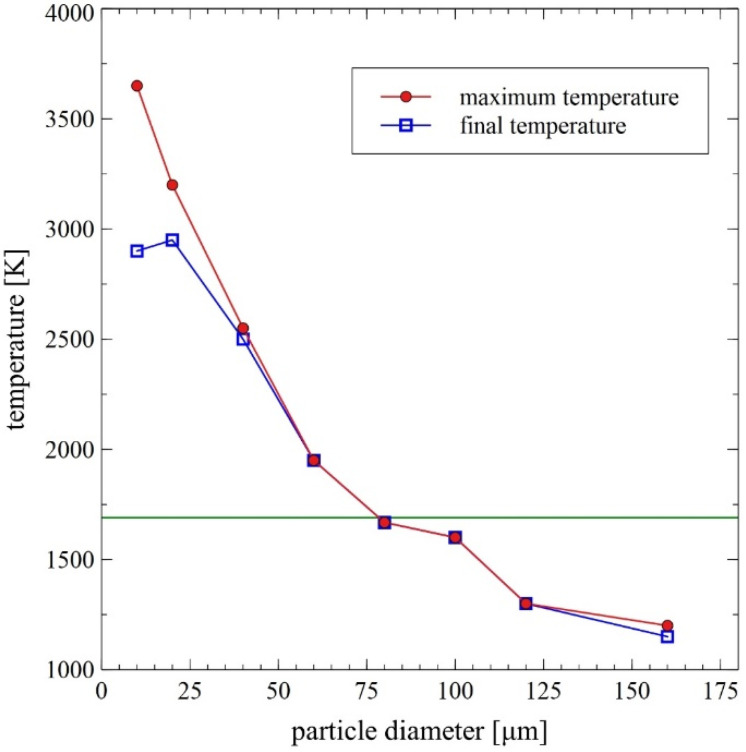
Comparison of the maximum and final particle temperature during the DGS spraying process as a function of the particle diameter—results of the DGS multiphase flow numerical modeling [14] with indication of the temperature of FeAl alloy melting.

**Figure 8:**
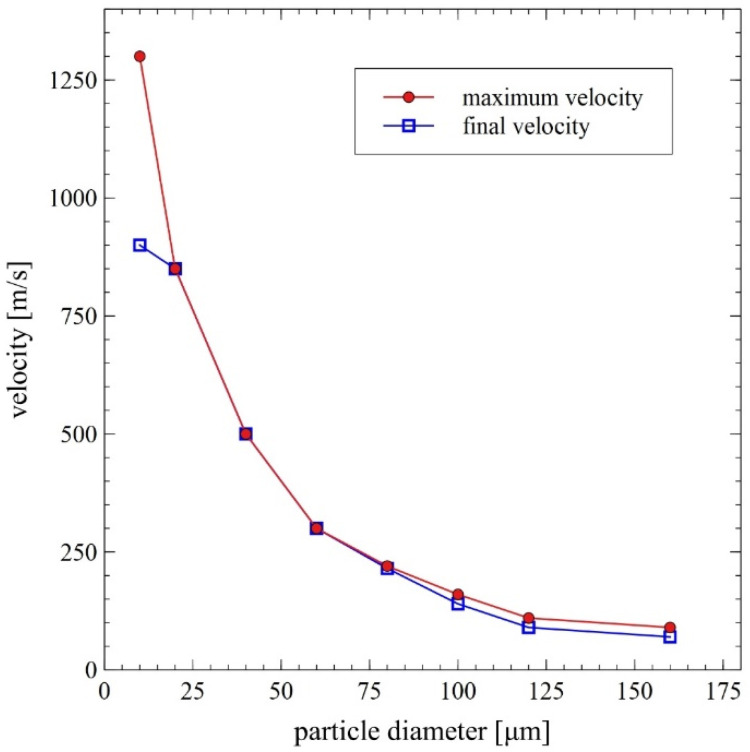
Maximum and final (at the base surface, i.e., water level) particle velocity as a function of its diameter—results of the DGS multiphase flow numerical modeling [14].

In the course of numerical calculations, the critical diameter for unmelted particles was established to be equal to about 80 µm [14]. Collective results of the numerical analyses are presented in Figure 7 and Figure 8.

The analysis of the presented data confirms the exceptionally large range of the thermodynamic state of the sprayed particles, which is a distinguishing feature of the gas detonation spraying process. On the one hand, it presents a great difficulty in planning an experiment and developing a DGS technology. On the other hand, it could be used to specifically shape the properties of the coating. It should be highlighted, however, that this is certainly the main factor differentiating the internal structure of the FeAl coatings [8,18,24,25], created by melted or unmelted particles depending on the heat transfer efficiency resulting mainly from the dynamics of the DGS process. As reported by Senderowski et al. [8,18,24,25], the evident composite-like morphology of the FeAl type coating is related to the presence of dispersed intermetallic phases in the various stages of ordering (depending on the Al content). This effect is due to the considerable oxidation in the DGS process, which is the reason for the occurrence of the stable layers of oxides such as α-Al_2_O_3_ identified as thin films between the nanocrystalline grains of a dual-phase FeAl and Fe_3_Al structure.

## 4. Verification of Model Calculations for Gas Detonation Parameters by DGS Spraying of FeAl Powder Particles into Water

The experimental verification of the model of heat exchange between FeAl particles and the gas flowing around them was based on spraying such particles into water. The model validation included size and chemical composition of powder particles and their susceptibility to melting and deformation under specific DGS process conditions (presented in Section 2).

**Figure 9:**
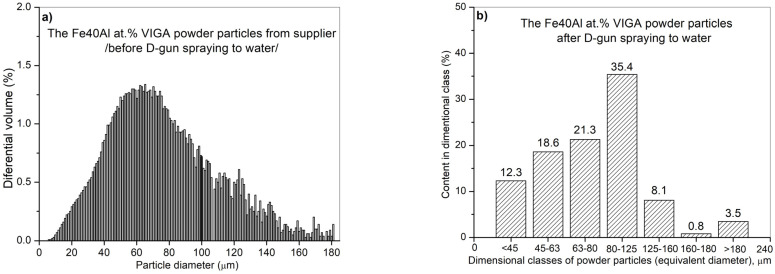
Analysis of the particle diameter distribution of the Fe40Al at.% (VIGA) powder using an IPS UA analyzer in as-received state: volumetric fraction (**a**), fraction of particles in specific size classes (**b**).

**Figure 10:**
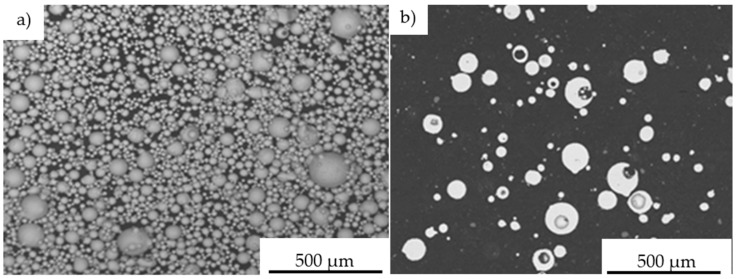
Morphology of Fe40Al at.% powder particles used in D-gun spraying into water: the dimension heterogeneity of the spherically structured particles (**a**), the cross-section reflecting the porosity of FeAl particles in their volume (**b**).

The performed IPS UA analysis and SEM/BSE observations of FeAl (VIGA) powder in the as-received state confirmed that it is characterized by spherical particles of various sizes in the range of approx. 5–180 µm (Figure 9 and Figure 10). The particle size distribution for each size class identified in the IPS UA study is presented in Figure 9b.

The largest volume of approx. 30.2% of particles with a size of 80 to 125 µm was identified after the test. At the same time, approx. 1.6% by volume share of particles larger than declared by the manufacturer was also found in powder mixture (160–180 µm). It should be mentioned that only approx. 7.5% of FeAl powder particles (from approx. 320,000 tested particles) possess a diameter in the range from 125 to 160 µm and as much as approx. 60.7% diameter in the range from 5 µm to 80 µm. Finally, the share of the smallest of particles from 5 to 45 µm was approx. 16.6% by volume.

**Figure 11:**
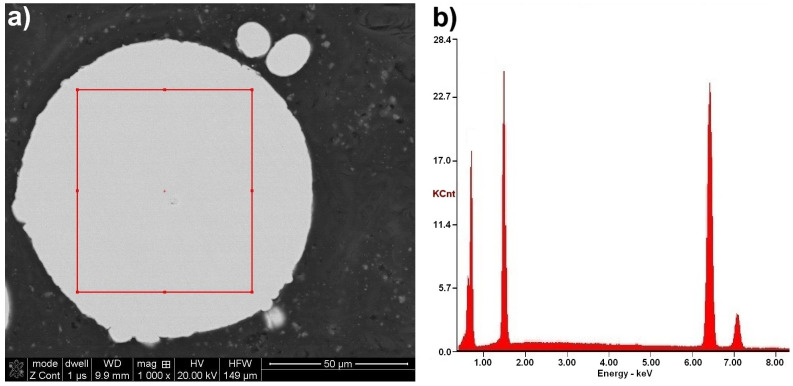
Typical FeAl solid solution microstructure on the cross-section of the FeAl VIGA powder particle (**a**), and SEM/EDX spectrum corresponding to the EDX area analysis (**b**).

Microscopic observations on the powder particles cross-sections confirmed their high dimensional heterogeneity. These particles were characterized by a spherical shape and high porosity (Figure 11). Such porosity results from the manufacturing process that includes the spraying of the FeAl alloy in an inert gas (argon) previously melted in a VIGA process (vacuum induction melting and inert gas atomization). Detailed investigations on particle porosity showed that the most numerous population (about 22% of 324 identified pores in 250 tested powder particles) were identified as pores with a size in the range of 20–30 µm [8].

The chemical composition microanalysis performed on the cross-sections of FeAl VIGA powder particles in an as-received state confirmed their homogeneity (Figure 11). Their structure was found as a secondary solution containing approx. 40 at.% aluminum, corresponding to the FeAl phase (Table 6).

**Figure 12:**
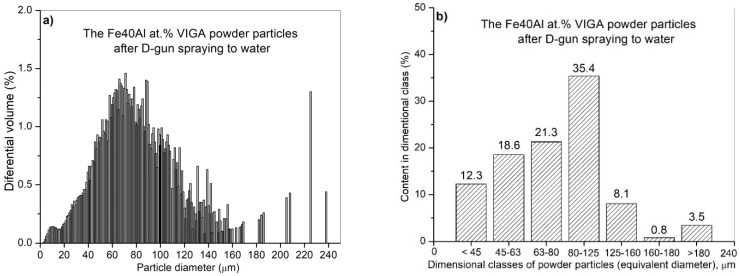
Analysis of the particle diameter distribution of the Fe40Al at.% (VIGA) powder using an IPS UA analyzer after state: volumetric fraction (**a**), fraction of particles in specific size classes (**b**).

**Figure 13:**
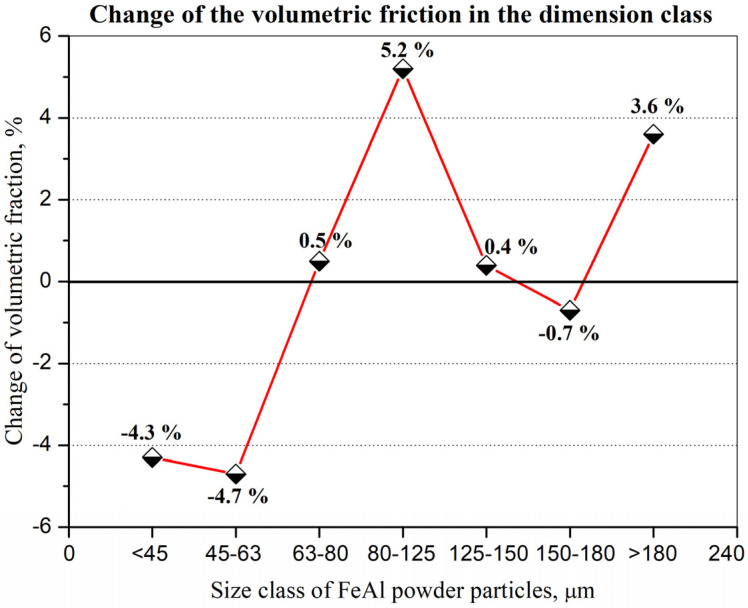
Percentage change in volume fraction in a specific size class of FeAl powder particles sprayed with D-gun into water as compared to as-received FeAl powder.

IPS UA dimensional analysis of FeAl particles sprayed under DGS conditions into water (Figure 12) showed some very large particles with diameter dimension over 180 µm (approx. 3.6%—Figure 13), which did not previously exist in the feedstock, were produced after D-gun spraying. These particles were not found in the as-received powder material (Figure 10). It was found that spraying FeAl particles into water under specific conditions of the DGS process led to an approx. 5.2% increase in the proportion of particles in the 80–125 µm range, with a simultaneous approx. 4.3% decrease in fine particles below 45 µm (Figure 13). A similar decrease of approx. 4.7% was also found in the case of particles in the 45–63 µm range.

**Figure 14:**
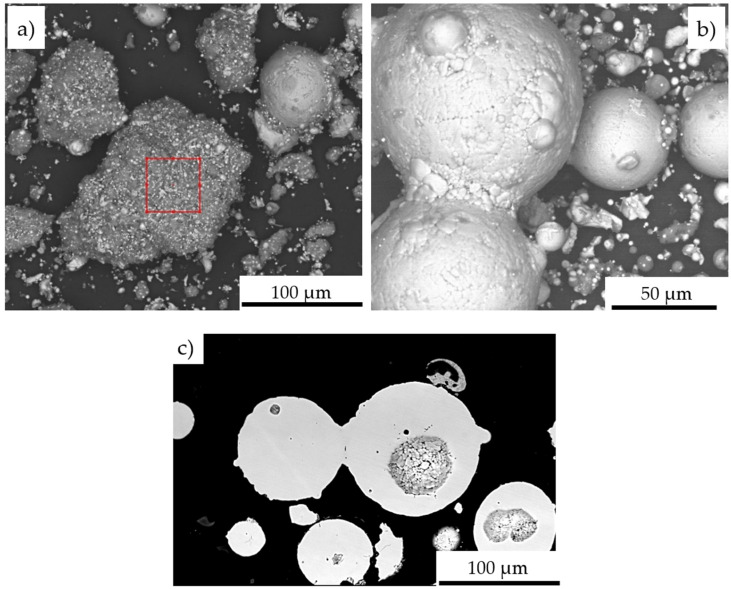
Morphology of Fe40Al at.% powder particles after DGS spraying into water: agglomerate formed from melted particles with the EDS analysis area marked (**a**); welding of two larger FeAl powder particles with larger dimensions (**b**); the cross-section of the FeAl particles welded together with the pores in the particle volume (**c**).

The SEM/EDS observations reveal, that the change in particle size distribution could be related to the melting of powder particles below 60 µm, which form larger grain clusters upon impact with the water surface (Figure 14). Created agglomerates were formed by the welding of molten fine particles dispersed in a gas stream (Figure 14a). As a result of phase transformations of the FeAl phase melting in the stream of hot gases in the DGS process, larger-size FeAl powders, melted on the surface, can also be welded (Figure 14b,c). This may explain the formation of about 3.6% of FeAl powder particles of diameter above 180 µm, which were not observed in the powder charge introduced into the “Perun-S” detonation gun feeder.

The chemical composition analysis from the surface of five randomly selected grain clusters, formed from melted particles of DGS powder sprayed into water (Figure 14a), presents a significant increase of aluminium and oxygen contents, which due to the lack of SEM/EDS calibration should be treated as a qualitative—semi-quantitative analysis (Table 7).

The high oxygen content with a significant increase in aluminum content on the surface of agglomerates formed from melted FeAl powder particles confirmed the formation of oxide phases under the DGS conditions. The privileged zones of in situ formation of thin Al_2_O_3_ films in the structure of gas detonation sprayed FeAl coatings (which are also partially morphized) are presented in other works by one of the authors [8,24,25].

## 5. Conclusions

In this work, the effect of thermo-kinetic conditions of the gaseous detonation stream on the heating degree and susceptibility to melting of FeAl powder particles during their spraying into water under specific conditions of the DGS process was determined analytically and numerically.

Experimental and modelling analysis on the mechanisms of heat transfer by FeAl powder particles in the stream of gaseous products of detonation combustion, for the DGS spraying process of powder particles into water, enables the following conclusions to be drawn:

1. Gas parameters and thermodynamic state of combustion products at the time of a detonation explosion of the gas mixture in the C-J plane is sufficient to melt FeAl powder particles up to 5 µm in diameter;
2. Under conditions of thermal forcing in the C-J plane, when shock wave passes through the powder particles, the changes in particle velocity are much faster than the particle temperature changes caused by convective heating. Therefore, the main factor affecting the heating and melting degree of FeAl powder particles are gaseous combustion products, detonation during their adiabatic expansion after shock wave passing;
3. Thermal diffusivity of Fe40Al at.% powder particles with a diameter of up to 60 µm (mainly determined by the thermophysical properties of the FeAl phase) does not constitute any barrier to reaching the temperature in the particle volume in accordance with temperature changes on its surface;
4. Powder particles with a diameter of 40 µm will not be able to absorb enough heat to melt them for the determined thermo-gas-kinetic parameters of the gas-detonation stream and the thermo-physical properties of the Fe40Al at.% intermetallic powder charge. Such considerations include the heat conduction in the volume of the FeAl particle and the radiation effects of heat exchange between the FeAl particle and the walls of the detonation gun barrel. On the other hand, for numerical calculations, this diameter is equal to 80 µm;
5. SEM microstructural characterization and particle size distribution of FeAl particles after the DGS spraying process into water revealed an approx. 5.2% increase in the proportion of particles in the 80–125 µm range, and a simultaneous approx. 4.3% decrease in fine particles below 45 µm. A similar decrease of approx. 4.7% was also found in the case of particles in the 45–63 µm range;
6. The analytical model predicts that approximately 16.6% of all particles melt, while the numerical model predicts that nearly 61% of all particles are subjected to melting.

## Figures and Tables

**Table 1:** DGS conditions for the 5–160 μm particles size of the Fe40Al at.% powder sprayed into water.

Detonation Gun Spraying Conditions	
Fuel gas (propane—C3H8)	0.45 m^3^/h
Oxidant (oxygen)	1.52 m^3^/h
Diluter gas (air)	0.25 m^3^/h
Carrier gas (air)	0.4 m^3^/h
Detonation frequency	6.66 Hz
PIP (powder injection position from ignition chamber)	275 mm
Spraying distance	110 mm

**Table 2:** Composition of detonation combustion products at DGS spraying of the Fe40Al at.% powder particles.

Working Explosive Gas Flow Rate [m^3^/h]: C_3_H_8_—0.45; O_2_—1.52; Air—0.25						
Detonation Blend	Concentration		Fraction			(mol/1 Working Cycle in the DGS Process)
	(mol/h)	(g/h)	(% vol.)	(% mas.)	(% mol.)	
1	2	3	4	5	6	7
C_3_H_8_	20.08	883.34	20.30	26.20	20.30	0.84 × 10^−3^
O_2_	70.15	2444.76	70.80	66.50	70.80	2.93 × 10^−3^
N_2_	8.71	243.94	8.80	7.20	8.80	0.36 × 10^−3^
Ar	0.10	4.16	0.10	0.10	0.10	0.43 × 10^−5^

**Table 3:** Thermo-kinetic parameters of combustion products at the time of detonation in the Chapman–Jouget (C-J) plane determined using the thermodynamic code TIGER.

Parameter	Value
a (m/s)	1425
D (m/s)	2614
u (m/s)	1188
$\rho \left(\frac{\mathrm{kg}}{m^{3}}\right)$	2.723
p (MPa)	4.444
T (K)	4461
$\kappa =\frac{c_{p}}{c_{v}}$	1.277
$\frac{\rho \cdot u^{2}}{2}$ (MPa)	1.92

a—speed of sound in the C-J plane. D—detonation velocity; u—mass velocity of detonation combustion products; *ρ*—density of gas detonation products; p—pressure; T—temperature; κ—isentropy exponent; $\frac{\rho .u^{2}}{2}$—kinetic energy of the gas detonation products.

**Table 4:** Chemical composition of gaseous products determined using the TIGER thermodynamic code from the balance of chemical equilibrium of detonation combustion of a mixture with the composition presented in Table 2.

Product	Molar Fraction z_i_, %	Mass Fraction g_i_,%
H_2_O	35.4997	29.8767
CO	33.6407	44.0411
H_2_	15.3888	1.4390
CO_2_	4.4557	9.3105
N_2_	4.3309	5.6699
O_2_	4.1090	6.1479
NO	2.5032	3.5111
NO_2_	0.0017	0.0037
NH_3_	0.0002	0.0002

**Table 5:** Average results of the model calculated thermal-physical parameters for two characteristics temperature ranges corresponding to solid and liquid state of aggregation of the Fe40Al at.% powder material and the results of parameters of FeAl phase changes of melting and evaporation.

Parameter	Solid	Liquid
	0−1395 °C	1395−2690 °C
Density *ρ_d_*, kg/m^3^	5560	4806
Specific heat *c_pd_*, J/(kg × K)	730	890
Thermal conductivity *λ_pd_*, W/(m × K)	15	71
Thermal diffusivity *a_d_*, m^2^/s	3.71 × 10^−6^	16.6 × 10^−6^
Surface emissivity *ε_d_*	0.7	
Melting enthalpy ∆*h_top_*, kJ/kg	288	
Evaporation enthalpy ∆*h_par_*, kJ/kg	7364	

**Table 6:** Chemical composition of FeAl VIGA powder particles.

Element	Content of the Elements	
	wt.%	at.%
Fe	24.72	40.46
Al	75.28	59.54

**Table 7:** Average results of SEM/EDS chemical composition tests on the surface of agglomerates formed from melted FeAl powder particles under specific DGS spraying conditions.

	Content of the Elements	
	wt.%	at.%
Fe	21.7	9.3 ± 7.2
Al	49.3	45.7 ± 4.1
O	~29	~45 ± 3

## Data Availability

The data are available in a publicly accessible repository.
